# Calibration of an Adaptive Genetic Algorithm for Modeling Opinion Diffusion

**DOI:** 10.3390/a15020045

**Published:** 2022-01-28

**Authors:** Kara Layne Johnson, Nicole Bohme Carnegie

**Affiliations:** Department of Mathematical Sciences, Montana State University, Bozeman, MT 59717, USA

**Keywords:** genetic algorithm, hyperparameters, control parameters, opinion diffusion, parameter estimation, social networks

## Abstract

Genetic algorithms mimic the process of natural selection in order to solve optimization problems with minimal assumptions and perform well when the objective function has local optima on the search space. These algorithms treat potential solutions to the optimization problem as chromosomes, consisting of genes which undergo biologically-inspired operators to identify a better solution. Hyperparameters or control parameters determine the way these operators are implemented. We created a genetic algorithm in order to fit a DeGroot opinion diffusion model using limited data, making use of selection, blending, crossover, mutation, and survival operators. We adapted the algorithm from a genetic algorithm for design of mixture experiments, but the new algorithm required substantial changes due to model assumptions and the large parameter space relative to the design space. In addition to introducing new hyperparameters, these changes mean the hyperparameter values suggested for the original algorithm cannot be expected to result in optimal performance. To make the algorithm for modeling opinion diffusion more accessible to researchers, we conduct a simulation study investigating hyperparameter values. We find the algorithm is robust to the values selected for most hyperparameters and provide suggestions for initial, if not default, values and recommendations for adjustments based on algorithm output.

## 1. Introduction

Genetic algorithms, developed by John Holland in the 1970s, mimic the process of natural selection to solve optimization or search problems and are particularly useful when the objective function lacks continuity, differentiability, or convexity or has local optima on the search space [1-6]. These algorithms represent a solution to the optimization problem as a chromosome consisting of genes. The chromosomes undergo biologically-inspired operators, modifying the genes to identify progressively better solutions. Hyperparameters or control parameters govern the behavior of these operators; however, specifying these hyperparameters is a barrier to use of genetic algorithms, particularly for researchers outside of the field of machine learning who are applying genetic algorithms in their research [7,8].

We developed a genetic algorithm to fit DeGroot opinion diffusion models using limited data on small social networks specifically for use in network and social science research [9]. While there were existing algorithms for the closely-related Stochastic Opinion Dynamics Model (SODM), both the maximum-likelihood-based algorithm and the particle learning algorithm were developed for online social networks and require far more data than are practical to obtain in the public health and social science applications for which we developed our method [10,11]. Though other bio-inspired algorithms may be viable options for fitting the DeGroot model–for example, bacterial foraging optimization (BFO) or particle swarm optimization (PSO)–these algorithms have a tendency to identify local as opposed to global optima: a concern motivating our choice of a genetic algorithm [12-14]. Using adaptive modifications of these algorithms, such as the self-adaptive chemotaxis strategy for bacterial foraging optimization (SCBFO), is a potential solution, but the performance of a genetic algorithm has already been demonstrated on the related problem of design of constrained mixture experiments with the structure of the design matrix and the matrix of parameters for the DeGroot model having a similar structure and the same sum-to-one constraint across rows [2,14]. Further, we demonstrated the performance of the genetic algorithm under the conditions expected in the intended application of this method [15].

While we adapted the operators from the genetic algorithm for design of mixture experiments, substantial changes to the operators were necessary due to model assumptions and the large parameter space relative to the design space. As such, the suggested hyperparameter values for the original algorithm cannot be expected to result in optimal performance. While research exists on either optimizing or removing hyperparameters from genetic algorithms in general, the algorithms and objective functions used bear even less resemblance to our algorithm because of the features specific to the opinion diffusion application [7,16]. To make the algorithm for modeling opinion diffusion more accessible to applied researchers, we conduct a simulation study investigating hyperparameter values, removing a barrier for researchers applying this methodological development to their applied research.

We begin by providing an overview of the model for opinion diffusion, detailing the genetic algorithm, and describing our approach and procedures for calibrating the algorithm in Section 2. We then present the results of the simulation study, addressing the performance of the algorithm in terms of parameter recovery and efficiency for the different hyperparameter values considered in Section 3. Finally, we conclude by tying the results back to the hyperparameters considered, providing specific suggestions for hyperparameter values and paying particular attention to hyperparameters that can negatively affect performance if poorly calibrated in Section 4.

## 2. Materials and Methods

In this section, first, we explain our model for opinion diffusion, focusing on the features that inform our decisions regarding the genetic algorithm. Then, we detail the genetic algorithm, highlighting the purposes of the operators and how the hyperparameters govern their behavior. Lastly, we describe the hyperparameters, procedures, and measures used in the simulation study.

### 2.1. Opinion Diffusion Modeling

In this section we provide an overview of the approach we take to opinion diffusion modeling. We focus on the details of the model to be fit and the modifications necessary to work with ordinal opinion data. Finally, we detail the objective function, highlighting why a new method was appropriate for optimization and why suggestions on hyperparameter values from the algorithm we adapted are not expected to be informative.

#### 2.1.1. DeGroot Model

The DeGroot model for opinion diffusion is a deterministic model that describes the process through which individuals or *agents* update their opinions from time *t,* through the influence of their social network contacts, to their opinions at time *t* + 1 using

(1) X(t+1)=WX(t),

where *X*(*t*) is an *N* × 1 vector of opinions and *W* is an *N* × *N* weight matrix. Each element in *X*(*t*), *x_i_*(*t*) ∈ [0, 1], represents the opinion of agent *i* at time *t* and each element of *W*, *w_ij_* ∈ [0, 1], represents the weight that agent *i* places on the opinion of agent *j* when updating their current opinion. The elements in *W* are restricted by $\sum_{j=1}^{N}w_{ij}=1$ so that *w_ij_* can be interpreted as the proportion of the total influence on agent *i* exerted by agent *j*. The model also incorporates the structure of the social network through an adjacency matrix *A*, where *a_ij_* = *a_ji_* = 1 if agents *i* and *j* can directly influence each other and *a_ij_* = *a_ji_* = 0 otherwise, with a link between *i* and *j* in the social network being the simplest definition of “ability to influence”. The adjacency matrix constrains the weight matrix by forcing *w_ij_* ≤ *a_ij_*, so the absence of influence implies zero weight. Though atypical for social network analysis, we include a self-link (*a_ii_* = 1) so that agents update their opinions based on their own current opinions.

#### 2.1.2. Transformations

The purpose of our method is to fit the above model, producing estimates for the parameters in the weight matrix *W*, using observed opinions across *T* time steps on a network of *N* agents. Ideally, we would be able to observe the continuous opinions, on the interval [0, 1], of all agents across *T* time steps (*X*(0), *X*(1),…, *X*(*T* – 1)). In practice, opinions are typically measured using a Likert or other ordinal scale in behavioral and social science research. As such, we assume the continuous opinions are shared with network contacts without error according to the DeGroot model, but researchers are only able to measure these opinions on an *n*-point ordinal scale (*Y*(0), *Y*(1),…, *Y*(*T* – 1)). To be consistent with the model, we assume these ordinal data possess interval properties. A common approach when using ordinal data, this is necessary to perform any mathematical operations and is implicit in the use of a composite scale. We convert between ordinal and continuous opinions using the following process:

##### Forward Transformation

1. Begin with data on an *n*-point ordinal scale, converting to a 1 to *n* scale if necessary.
2. Divide the interval [0, 1] into *n* sub-intervals of equal width.
3. An opinion of *y* on the ordinal scale takes on the middle value, *x*, in the *y^th^* sub-interval on the continuous scale.

##### Back Transformation

1. Begin with data on a continuous [0, 1] interval to be converted to an *n*-point ordinal scale.
2. Multiply the continuous opinion *x* by *n*.
3. Round the multiplied continuous opinion up to an integer (ceiling function) to produce an opinion on the ordinal scale. (This final step does not work for the edge case where *x* = 0, so any such values are automatically converted to an ordinal value of 1.)

**Figure 1.**
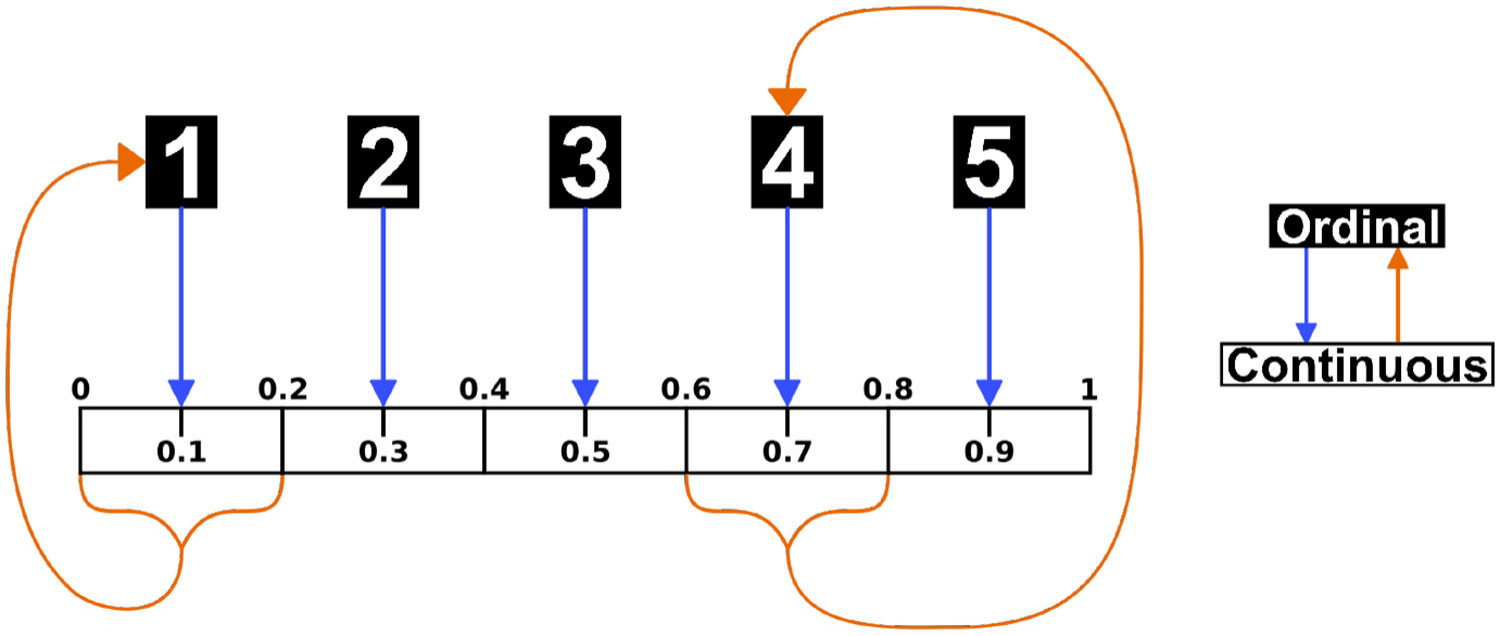
Transformation procedure for a 5-point ordinal scale.

This process is also presented graphically in Figure 1 using a 5-point ordinal scale. For example, an ordinal opinion of 4 is converted to a continuous opinion of 0.7, the center of the 4th sub-interval or *bin* from 0.6 to 0.8, and any continuous opinion on that sub-interval are converted back to an ordinal opinion of 4.

#### 2.1.3. Objective Function

Our selected objective function incorporates opinions on both the continuous and ordinal scales, accounting for an important feature of the back-transformation process: a range of continuous opinions map back to a single ordinal opinion. We use

(2a) $$f(\hat{X},X)=\sum_{i=1}^{N}\sum_{t=0}^{T-1}B\left({\hat{x}}_{i}(t),x_{i}(t)\right)|{\hat{x}}_{i}(t)-x_{i}(t)|,$$

where *N* is the number of agents in the network and $B({\hat{x}}_{i}(t),x_{i}(t))$ measures the absolute deviation between the observed and predicted opinions on the ordinal scale, measured in *bins*. This allows us to penalize deviation from the center of the correct interval on the continuous scale only if the observed and predicted opinions also differ on the ordinal scale $\left(B({\hat{x}}_{i}(t),x_{i}(t))\neq 0\right)$. Though this objective function is well-suited for our goal of fitting a model based on observed ordinal opinions, the inclusion of $B({\hat{x}}_{i}(t),x_{i}(t))$ presents problems for any optimization method requiring continuity or differentiability. We also expect many perfect solutions $\left(f(\hat{X},X)=0\right)$ will fail to recover the parameters, particularly for less precise ordinal scales (ones with fewer points) and fewer time steps. This objective function can also be assessed on a chromosome or agent level by excluding the sum across agents:

(2b) $$f({\hat{x}}_{i},x_{i})=\sum_{t=0}^{T-1}B\left({\hat{x}}_{i}(t),x_{i}(t)\right)|{\hat{x}}_{i}(t)-x_{i}(t)|,$$

which we leverage as part of the gene-swapping procedure in Section 2.2.1.

### 2.2. Genetic Algorithm

We use the genetic algorithm to identify the parameters of the DeGroot model, in the form of the weight matrix *W*, that minimize the objective function. A chromosome is defined as the weight matrix *W* and a gene as a row of *W*, denoted *W_i_* and representing the sources and strength of influence on agent *i*. We begin with a population consisting of an odd number of chromosomes, consistent with any fixed values. These fixed values are usually zeros resulting from zeros in the adjacency matrix (See Section 2.1.1) but can be other known parameters. Though the user has the option to specify chromosomes, the default is a population of randomly generated chromosomes and an identity matrix. This population undergoes selection, blending, crossover, mutation, and survival operators, incorporating a gene-swapping procedure, to identify an optimal solution.

#### 2.2.1. Gene Swapping

Since the objective function can be assessed on the individual– or gene–level, the fitness of a gene clearly does not directly depend on the other genes within the chromosome. Instead, the fitness of a gene *W_i_* depends on the predicted opinions of the agents who influence agent *i*, as indicated by non-zero elements within *W_i_*. Since these predicted opinions are a function of the genes corresponding to the agents who influence agent *i*, the fitness of a gene can be assessed independently of other genes within the chromosome but does depend those other genes. We use this ability to assess fitness at the gene level while accounting for dependencies in our gene-swapping process.

At any point in the algorithm where we identify the fittest chromosome, we assess the fitness of each gene within that chromosome and for all other chromosomes. If the overall fittest chromosome *B* contains a gene *B_i_* which is less fit than the corresponding gene *C_i_* in a less fit chromosome *C*, we swap *B_i_* and *C_i_* between the two chromosomes to produce B* and C*. We retain this change for both chromosomes if B* is fitter than *B* and revert to *B* and *C* otherwise. In the case where the less fit population contains multiple chromosomes, we swap all fitter genes at once, either retaining all swaps or reverting to the original chromosome *B*. This helps prevent the loss of a fit gene in an otherwise unfit chromosome while ensuring the best solution identified so far is retained.

#### 2.2.2. Operators

We apply selection, blending, crossover, mutation, and survival operators to our population of chromosomes. The application of all operators constitutes a single iteration of the algorithm and produces a new generation of chromosomes. We use “iteration” and “generation” interchangeably except where a distinction between the process of producing a new generation (in this case referred to as an iteration) and the generation itself is meaningful. We repeat the process until stopping criterion are met, modifying the behavior of the operators as the generations progress to shift from exploration of the parameter space to exploitation of existing solutions, making this an adaptive genetic algorithm as suggested by the literature [2,17-19]. Descriptions of the operators that include examples are available in Johnson et al. and the code is linked in the Data Availability Statement [9].

##### Selection:

In order to preserve the best solution identified in any previous generation, we use selection with elitism: identifying the fittest chromosome (the chromosome producing the lowest value of the objective function), and exempting it from the remaining operators until the next generation. After identifying the elite chromosome, we attempt gene swapping between that chromosome and the remaining population of non-elite chromosomes. We exempt either the original elite chromosome or row-swapped elite chromosome, depending on the fitness of each, and proceed with either the original remaining population or the row-swapped remaining population as appropriate.

##### Blending:

Using the even number of chromosomes remaining after the selection operator, we randomly pair all chromosomes. For each pair of chromosomes, blending occurs independently for each gene with probability *p_b_*. For a pair of chromosomes *B* and *C*, if blending occurs for row *i*, a blending factor *β* is drawn from a *Unif*(0,1) distribution. The new genes ($B_{i}^{*}$ and $C_{i}^{*}$) are the weighted averages of the current genes and corresponding genes from the paired chromosome according to:

(3) $$B_{i}^{*}=\beta B_{i}+(1-\beta )C_{i}\;\;\text{and}\;\;C_{i}^{*}=(1-\beta )\;B_{i}+\beta C_{i}.$$

While this can result in substantial changes to genes *B_i_* and *C_i_* when these genes are very different, we use the blending operator primarily to make slight changes to a population of similar chromosomes for later generations in order to refine a solution as we shift from exploration to exploitation, meaning we begin with a lower value of *p_b_* and increase the probability over time.

##### Crossover:

The within-parent crossover operator defined by Limmun, Borkowski, and Chomtee uses a crossover point after the decimal point, resulting in small changes to the genes [2]. Since both the blending and mutation operator either already accomplish this goal or can easily be modified in later generations to do so, we use a more drastic version of this operator to explore our much larger parameter space. Crossover occurs independently for each gene within each chromosome with probability *p_c_*, with all values not fixed at zero randomly reshuffled within the gene, preserving the sum-to-one constraint and any fixed values. Since exploring the parameter space is desirable during early generations, but drastic changes to chromosomes are not helpful in later generations, we begin with a higher value of *p_c_* which decreases over time.

##### Mutation:

While the mutation operator is also used to make slight changes to genes for later generations, the primary purpose of this operator is to explore the boundaries of the parameter space: solutions where a gene contains a weight where *w_ij_* = 1 and all others are zero (or with *w_ij_* = 1 – *w_fixed_* where *w_fixed_* is the sum of all fixed weights within the row). Since our method for generating the initial population of chromosomes–drawing each weight from a *Unif*(0, 1) distribution and scaling the rows to sum to one–will not result in any edge cases other than the identity matrix included in the initial population, this is necessary in order to consider edge cases as potential solutions. Mutation occurs independently for all genes within each chromosome with probability *p_m_*. If mutation occurs, *ε* is drawn from a *N*(0, *σ*^2^) distribution and added to a randomly selected weight within the gene to produce w* = *w* + *ε*, and all other non-fixed weights are scaled by $\frac{1}{1-w_{fixied}-w^{*}}$ to preserve the sum-to-one constraint. We handle edge cases as follows:

- If w* < 0, w* is set to 0, with scaling of other non-fixed weights as above.
- If w* > 1 – w_fixed_, w* is set to 1 – *w_fixed_*. All other non-fixed weights in the row are set to 0.
- If the selected weight *w* = 1 – *w_fixed_*, the excess weight of 1 – *w_fixed_* – w* is evenly distributed between all other non-fixed weights within the row.

##### Survival:

After the preceding operators, our population of chromosomes includes the elite chromosome, the parent chromosomes (the chromosomes from the previous generation), and the offspring chromosomes (the chromosomes from the current generation, having undergone the selection, crossover, and mutation operators). For each pair of parent and offspring chromosomes, we identify the fittest chromosome and attempt gene swapping with the other chromosome. The fittest chromosome from each pair after the attempted gene swapping along with the elite chromosome constitute the current generation and become the parent chromosomes for the next generation.

#### 2.2.3. Other Features

As discussed in the descriptions of the operators, we use an adaptive genetic algorithm where each operator becomes more or less important as the generations progress, and the mutation operator in particular can be modified to serve a different purpose. This allows us to begin with a focus on exploration of the parameter space and progressively move to refining existing solutions (exploitation). For the sake of clarity, we will refer to the values controlling behavior of the individual operators, the operator probabilities (*p_b_,p_c_,p_m_*) and *σ*, as *control parameters* and reserve the term *hyperparameters* for the user-specified values that govern the overall behavior of the algorithm, including the way the control parameters are modified within the algorithm. We modify the control parameters by applying a multiplicative adjustment whenever a specified number of generations without improvement is reached. For example, $p_{b}^{*}=cp_{b}$ for the specified constant *c* where $p_{b}^{*}$ is the new value of the probability of blending.

We apply a similar process with chromosome reintroduction: reintroducing either a clone of the elite chromosome or an identity matrix after a specified number of generations without improvement. Reintroducing a clone of the elite chromosome allows slight changes to the current best solution—facilitating exploitation—while still preserving this solution in the selection operator. Reintroducing an identity matrix reinforces a prior belief that agents place high weight on their own opinions. In either case, the reintroduced chromosome replaces the least fit chromosome in the population.

### 2.3. Algorithm Calibration

**Figure 2.**
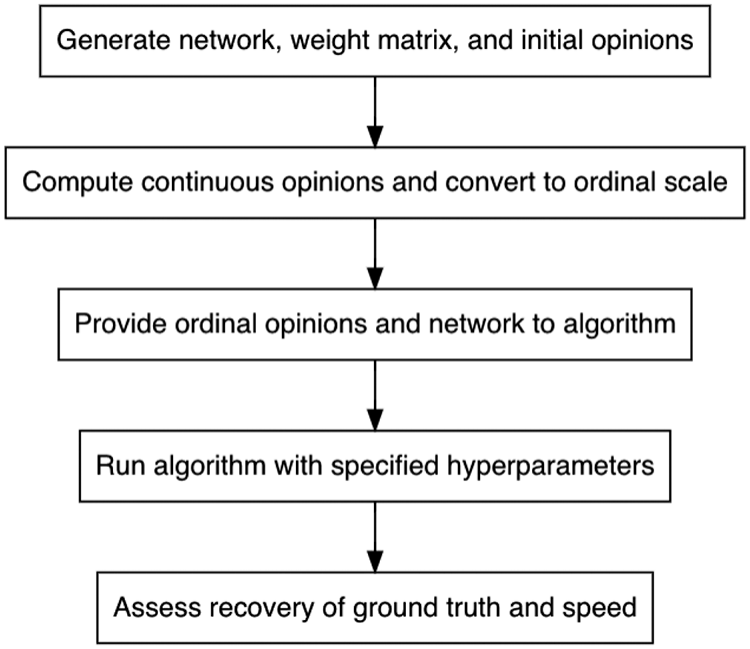
Procedure for algorithm calibration.

In this section, we explain all aspects of the simulation study to calibrate the algorithm, detailing the hyperparameters and our approach for condensing them into groups, describing the procedure for the simulation study, and presenting the measure used to assess algorithm performance. Though tuning approaches such as the Chess Rating System (CRS-tuning), Relevance Estimation and Value Calibration (REVAC), and F-race are available, the simulation study approach, overviewed in Figure 2, better suits our objectives [8,20]. The simulation study facilitates investigating the relationship between hyperparameter values and performance and providing accessible suggestions to algorithm users based on the results while acknowledging that both the relationship and suggestions may depend on network or dataset characteristics.

F-race identifies a set or sets of hyperparameters that are statistically significantly better than others; however, our goal is not to identify an ideal set of hyperparameters based on an arbitrary threshold but to characterize the behavior of the algorithm under various hyperparameter combinations [21]. While CRS-tuning does address the concerns of the binary include or exclude through the use of a ranking system, this raking does not contain the information necessary for users to develop intuition about how the different hyperparameters affect algorithm behavior [22]. Since REVAC identifies a marginal distribution of high-performing values for each hyperparameter that approximates the maximum Shannon entropy distribution, this approach produces a distribution of values instead of a single value, and the relevance of each parameter can be measured [23,24]. While these are both appealing, the simulation study allows for an assessment of relevance through the relationship between the values used and algorithm performance while also presenting this overall relationship in an accessible manner that incorporates network and dataset features.

#### 2.3.1. Hyperparameters

Table 1 contains all of the hyperparameters used in the algorithm. Since most are considered in the simulation study, we highlight the ones that are not: max_iter, min_improve, min_dev, and reintroduce. In all simulations, we run the algorithm until we either reach 100,000 iterations (max_iter=100,000) or identify a perfect solution on the ordinal scale (min_dev=0). Note that this check is only applied every thousand generations. We do not specify a minimum change in the objective function that is considered an improvement (min_improve=0) and reintroduce the elite chromosome (reintroduce=“elite”).

To reduce the simulation study to a manageable size, we condense some of these hyperparameters to a single value or otherwise group them. We consider 200, 1000, and 5000 generations without improvement before modifying control parameters or reintroducing a chromosome, using the same value for all relevant hyperparameters (iterb, iterc, iterm, iters, and iterr) within a run of the algorithm. The remaining hyperparameters are grouped into ProbSigma (probb, probc, probm, and sigma), MinMax (maxb, minc, minm, and mins), and MultFactor (factorb, factorc, factorm, factors). These groupings represent the starting values of the control parameters, the minimum or maximum values for the control parameters, and the factors for multiplicative adjustment to the control parameters, respectively. Since the hyperparameters within a group cannot reasonably be set to the same value, we instead define three levels for each group with differing values of each hyperparameter but consistent goals or concepts.

For ProbSigma, we use *low, medium*, and *high* to indicate whether we use low, medium, or high initial values for the control parameters. Table 2 shows the specific hyperparameter values corresponding to each level for ProbSigma. For MinMax, we use *minimal, moderate*, and *extreme* to specify whether we applied minimal, moderate, or extreme restrictions on the minimum or maximum value of each control parameter. The specific values corresponding to each level for MinMax are in Table 3. Note that the *minimal* level imposes no restrictions on the probabilities beyond those either implied as probabilities or possible through a multiplicative adjustment. Finally, we use *slow, moderate*, and *rapid* levels for MultFactor, indicating whether the multiplicitive factors used will result in slow, moderate, or rapid changes to the control parameters, with specific values for each level in Table 4.

#### 2.3.2. Procedure

The hyperparameters and features of the social network and opinion diffusion process considered in the simulation study are in Table 5. We use each combination for ten runs of the algorithm, generating a new network, weight matrix, and dataset each time. First, we generate an Erdős-Rényi network, to represent a cluster within a larger network, of the specified size and target degree, rejecting any disconnected networks (networks that do not include a path between every pair of agents). We generate a weight matrix using a target self-weight of *w_ii_* = 0.5, drawn from a beta distribution with *κ* = *α* + *β* = 4, and draw all other weights from a *Unif*(0, 1) distribution, scaling all weights other than the self-weight to maintain the sum-to-one constraint. Note that this approach results in a ground truth that is biased against edge cases, as is the population of initial chromosomes other than the identity matrix. Then, we draw initial opinions (*X*(0)) from a *Unif*(0,1) distribution, using these and the weight matrix to generate “true” opinions across the specified number of time steps (*X*(1),…, *X*(*T* – 1)). Finally, we convert the latent, continuous opinions to the appropriate ordinal scale to produce observed opinions (*Y*(0),…, *Y*(*T* – 1)), using the back-transformation process (See Section 2.1.2). We provide the adjacency matrix representing the generated network and the observed opinions to the algorithm, using the specified hyperparameter values.

#### 2.3.3. Measures

Optimal hyperparameters would quickly identify a perfect solution in terms of the objective function. Ideally, this perfect solution would also result in good parameter recovery. Since how quickly the algorithm identifies a solution can be measured in both number of generations and time, we record the amount of time and the number of generations to reach a solution, both measured in thousand-generation increments. Simulations were run on a custom desktop with a Ryzen 9 3950X CPU with 64 GB of 3000 MHz RAM on Ubuntu Server 21.10 and Julia 1.5—using a single thread per run of the algorithm—and use @elapsed to time in thousand-generation increments [25]. We assess parameter recovery using root-mean-square-error (RMSE) according to

(4) $$RMSE_{rec}=\sqrt{\frac{\sum_{i=1}^{N}\sum_{j=1}^{N}(w_{ij}-{\hat{w}}_{ij}{)}^{2}}{\sum_{i=1}^{N}\sum_{j=1}^{N}a_{ij}}}=\sqrt{\frac{\sum_{i=1}^{P}(w_{p}-{\hat{w}}_{p}{)}^{2}}{P}},$$

where *P* is the number of elements not fixed at zero in the weight matrix (the number of parameters to be estimated) and *w_p_* is the *p^th^* non-structurally-zero element, with *w_p_* and ${\hat{w}}_{p}$ representing the true and estimated weights, respectively. Though we also assessed the ability of the algorithm to model the latent opinions on the observed time steps and predict future latent opinions in Johnson et al., parameter recovery implies the other outcomes and is not possible to measure in practical applications [15]. As such, selecting hyperparameters that improve parameter recovery is our priority.

## 3. Results

To provide context for this section, note that the existence of a perfect solution is guaranteed (the ground truth used to generate the data) but many “perfect” solutions that fail to recover the parameters are expected, particularly for runs with imprecise ordinal scales (i.e., few bins) and few time steps. Overall, the algorithm identified a perfect solution very quickly regardless of the hyperparameters used, with 67.7% of runs finding a solution within the first 1000 generations. Only 4.5% of runs failed to identify a perfect solution within 100,000 generations, though the largest value of the objective function for these runs was 0.02, representing a good—but imperfect—solution. Since we prioritize recovery over either measure of speed, we begin by assessing the hyperparameters that produce the best recovery. Informed by the results on recovery, we assess speed in number of iterations, the measure independent of the computer used. Finally, we present results on computation time to provide context on the trade-off between time per generation and number of generations.

### 3.1. Parameter Recovery

**Figure 3.**
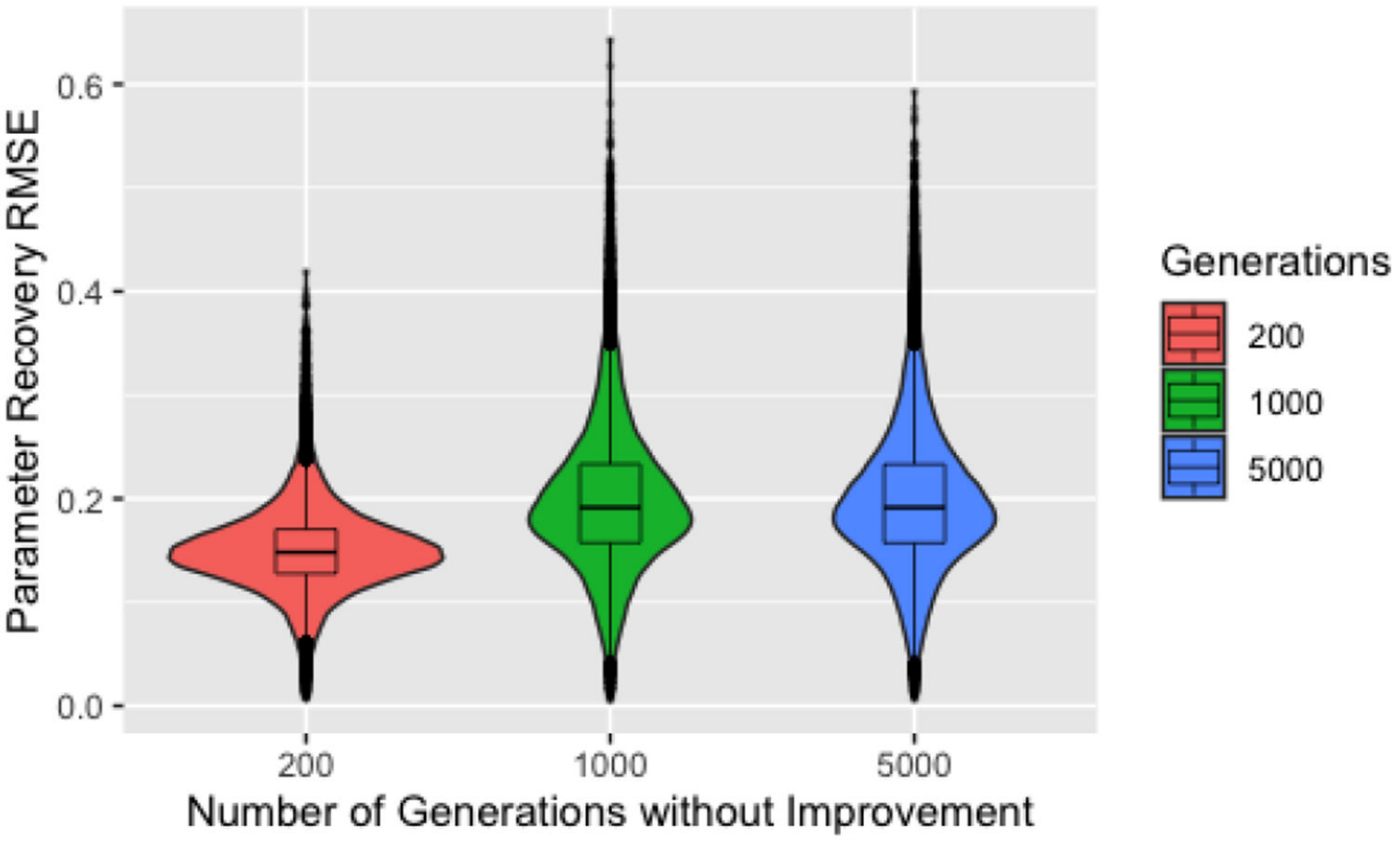
Boxplots and violin plots for root-mean-square-error for recovery by number of generations without improvement for runs that identified a solution with 1000 generations.

Figure 3 shows parameter recovery RMSE by number of generations without improvement before the control parameters are modified and the elite chromosome reintroduced, the only set of hyperparameters that produces a notable difference in parameter recovery. This plot includes only the subset of the data where a perfect solution was identified within the first 1000 generations to illustrate our next point, but the features seen in this plot hold for the full dataset. Clearly, using 200 generations without improvement results in the best parameter recovery. Nothing that the populations of chromosomes requiring either 1000 or 5000 generations without improvement could not possibly have begun the exploitation phase within 1000 generations, this initially seems intuitive since we would expect solutions resulting from the exploitation phase to be better. Unfortunately, this does not explain the results since all the solutions presented here are perfect in terms of the value of the objective function. Instead, we must explain why perfect solutions identified during the exploration phase have worse recovery that perfect solutions identified during the exploitation phase.

**Figure 4.**
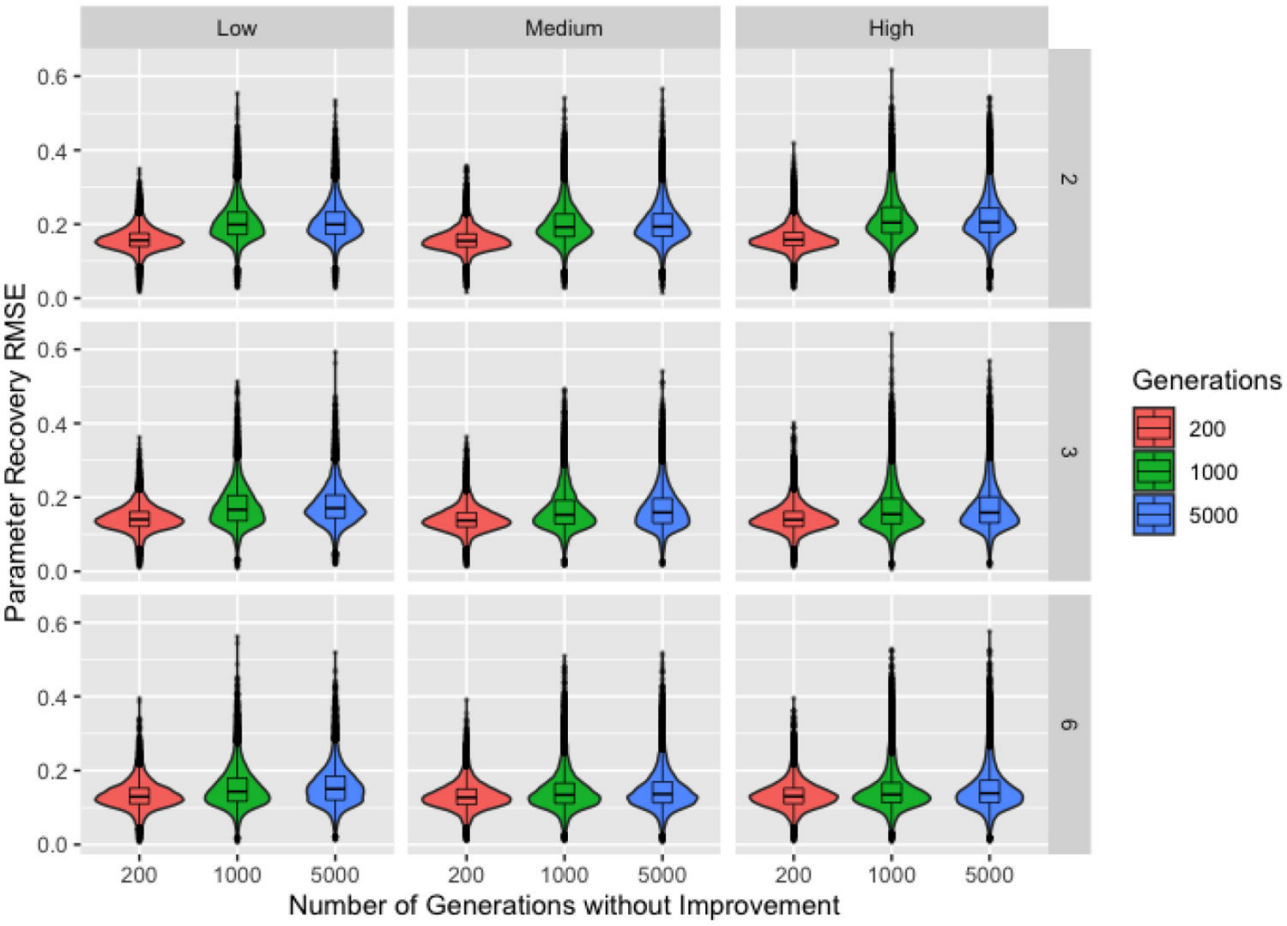
Boxplots and violin plots for root-mean-square-error for recovery by number of generations without improvement with ProbSigma hyperparameter levels (horizontal) and number of time steps (vertical) across facets.

To do so, we revisit the intended purpose of the exploration process. During early iterations of the algorithm, we use control parameter values that result in drastic changes to the chromosomes and force—to varying degrees depending on the ProbSigma hyperparameters—the exploration of edge cases. As noted in our description of the procedures, our process for generating the true parameters is biased against edge cases. Consequently, populations forced to search the boundaries of the parameter space will identify solutions with poor recovery. Figure 4 supports this assertion by showing parameter recovery for 200, 1000, or 5000 generations without improvement by level of ProbSigma and number of time steps. For the purpose of transparency, this plot uses the full dataset. We use the number of time steps as a proxy for the prevalence of perfect solutions in the parameter space and include ProbSigma as it governs the extent to which early generations are forced to explore edge cases (The precision of the ordinal scale is also indicative of the ease of finding a perfect solution and demonstrates the same phenomenon seen in Figure 4. We selected number of time steps because the fewer levels of that factor improve readability of the plot).

Comparing across number of time steps, we see the difference in parameter recovery between different numbers of generations without improvement decreases as the number of time steps increases (decreasing the number of potential perfect solutions). This supports our assertion that the poor parameter recovery is the result of forcing the algorithm to search the boundaries, where any solutions identified will inherently result in poor recovery. When an increased number of time steps makes it more difficult to identify an edge-case solution, the threshold for number of generations without improvement can then be reached, starting the transition away from the exploration phase and pulling the chromosomes away from the boundary. As expected, high values for the hyperparameters in the ProbSigma group—corresponding to the *high* level—appear to exacerbate this difference since larger values of the control parameter *σ* apply stronger pressure to search the boundaries. It is much more difficult to assess any differences between the *low* and *medium* levels, but the level of ProbSigma also controls the values of *p_b_*, *p_c_*, and *p_m_*, any of which could have a moderating effect on the value of *σ*.

### 3.2. Generations

**Figure 5.**
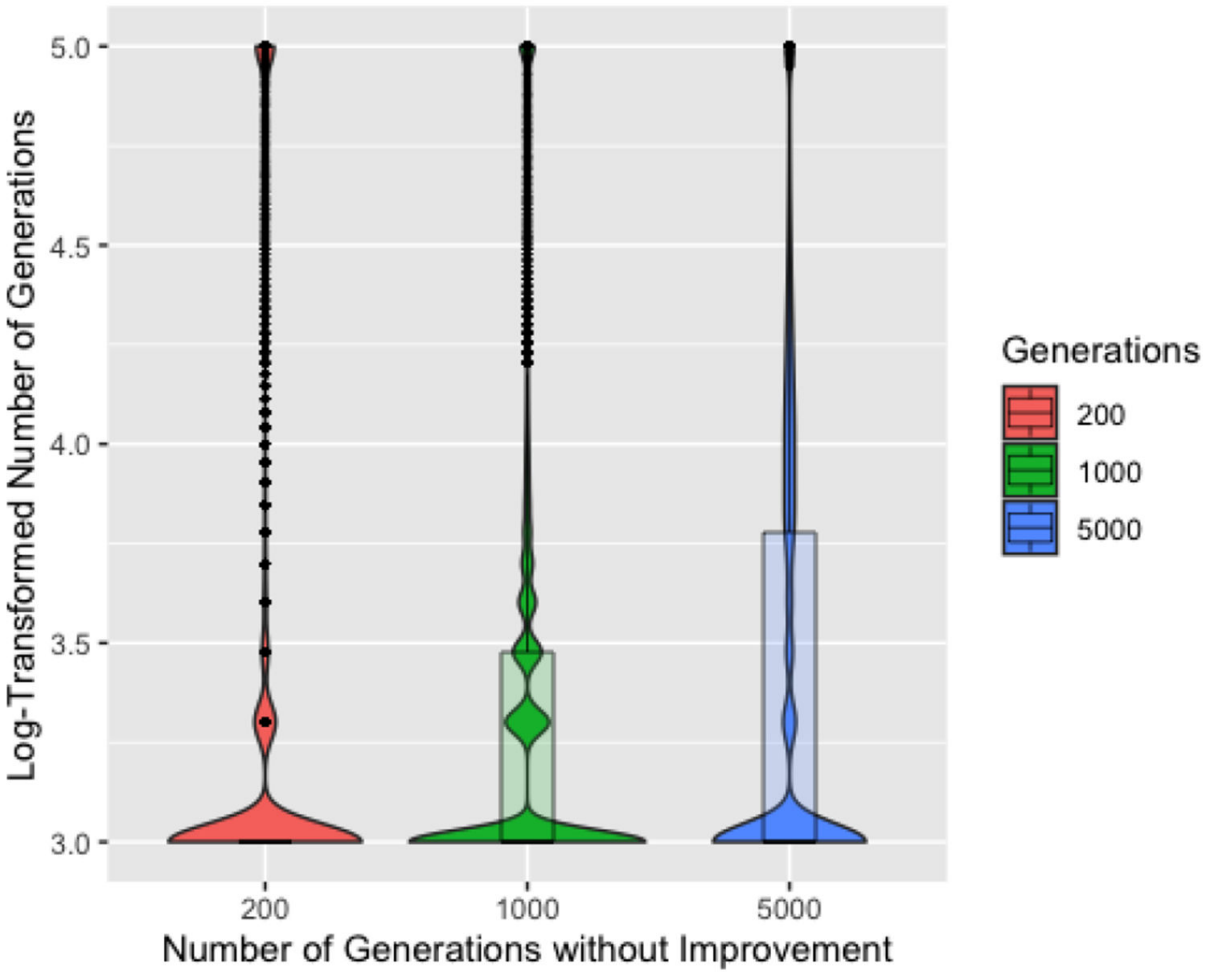
Boxplots and violin plots for log (base 10) number of generations to solution by number of generations without improvement. The absence of a box for 200 generations without improvement indicates that the median, first quartile, and third quartile are the same.

While the poor parameter recovery with 1000 or 5000 generations without improvement when the ground truth is biased against edge cases does not necessarily imply they will perform poorly in practical applications, having $\frac{2}{3}$ of our runs identify a solution within 1000 generations does suggest lower values may be a better choice. Figure 5, showing the log-transformed number of generations to a solution by number of generations without improvement, further supports that 200 generations is a better choice. 200 generations consistently requires the fewest generations necessary to find a solution, though it also has the highest density of runs requiring 100,000 generations, suggesting a slight tendency to transition from the exploration phase too quickly and become stuck near a local minima that is not a perfect solution. We will consider only 200 generations without improvement for the remainder of these results.

**Figure 6.**
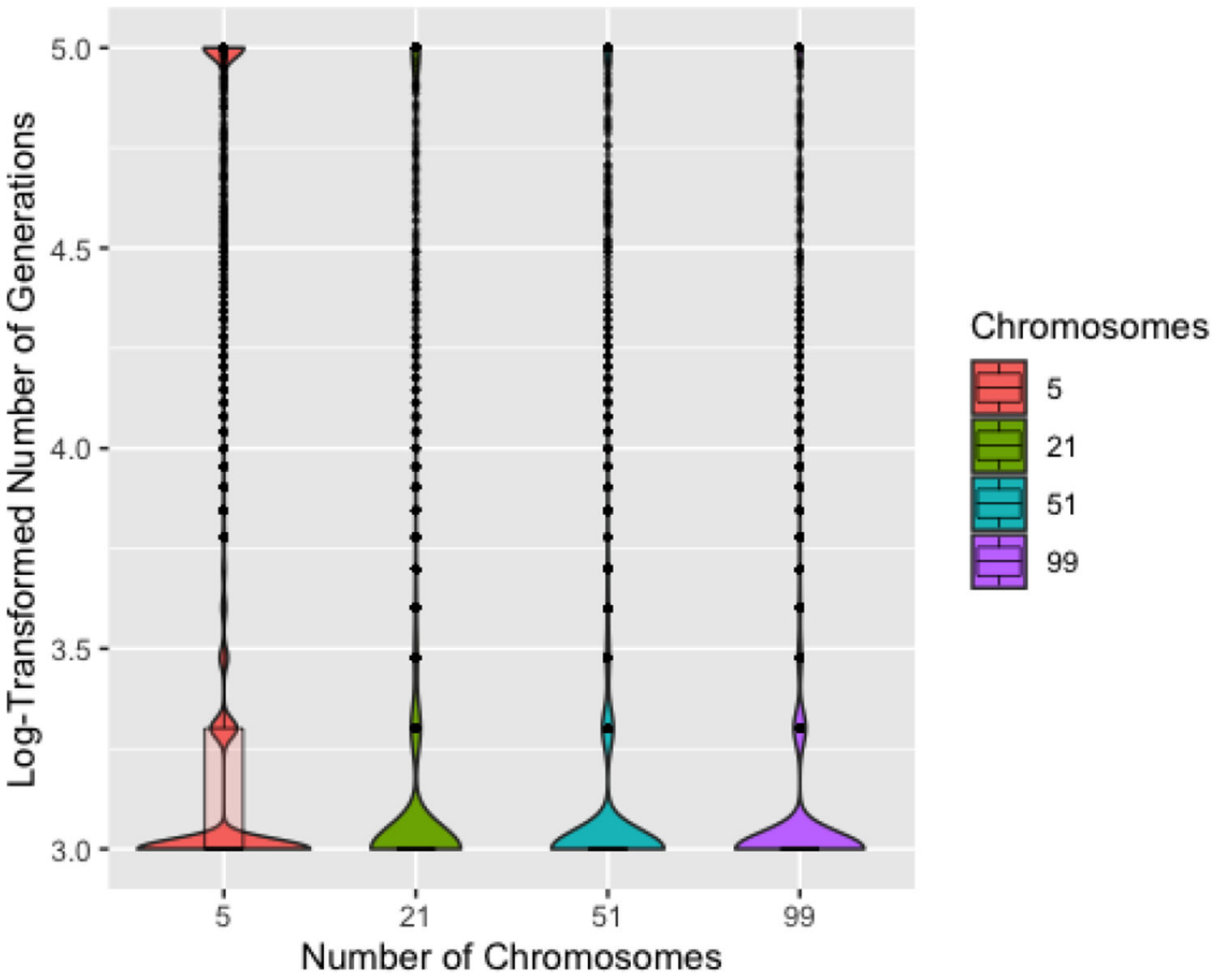
Boxplots and violin plots for log (base 10) number of generations to solution by number of chromosomes for 200 generations without improvement. The absence of a box for 21 or more chromosomes indicates that the median, first quartile, and third quartile are the same.

Figure 6 shows the log-transformed number of generations to a solution by number of chromosomes. Unsurprisingly, five chromosomes typically requires more generations to identify a solution and also has the most runs reaching 100,000 generations. Though each iteration would be completed more quickly with only five chromosomes, the iterations are much less efficient. Five chromosomes also resulted in slightly worse recovery overall, though this difference is barely discernible in a plot, so we remove five chromosomes from consideration. ProbSigma, MinMax, and MultFactor all showed minimal difference in number of iterations to find a solution across the varying levels.

### 3.3. Time

**Figure 7.**
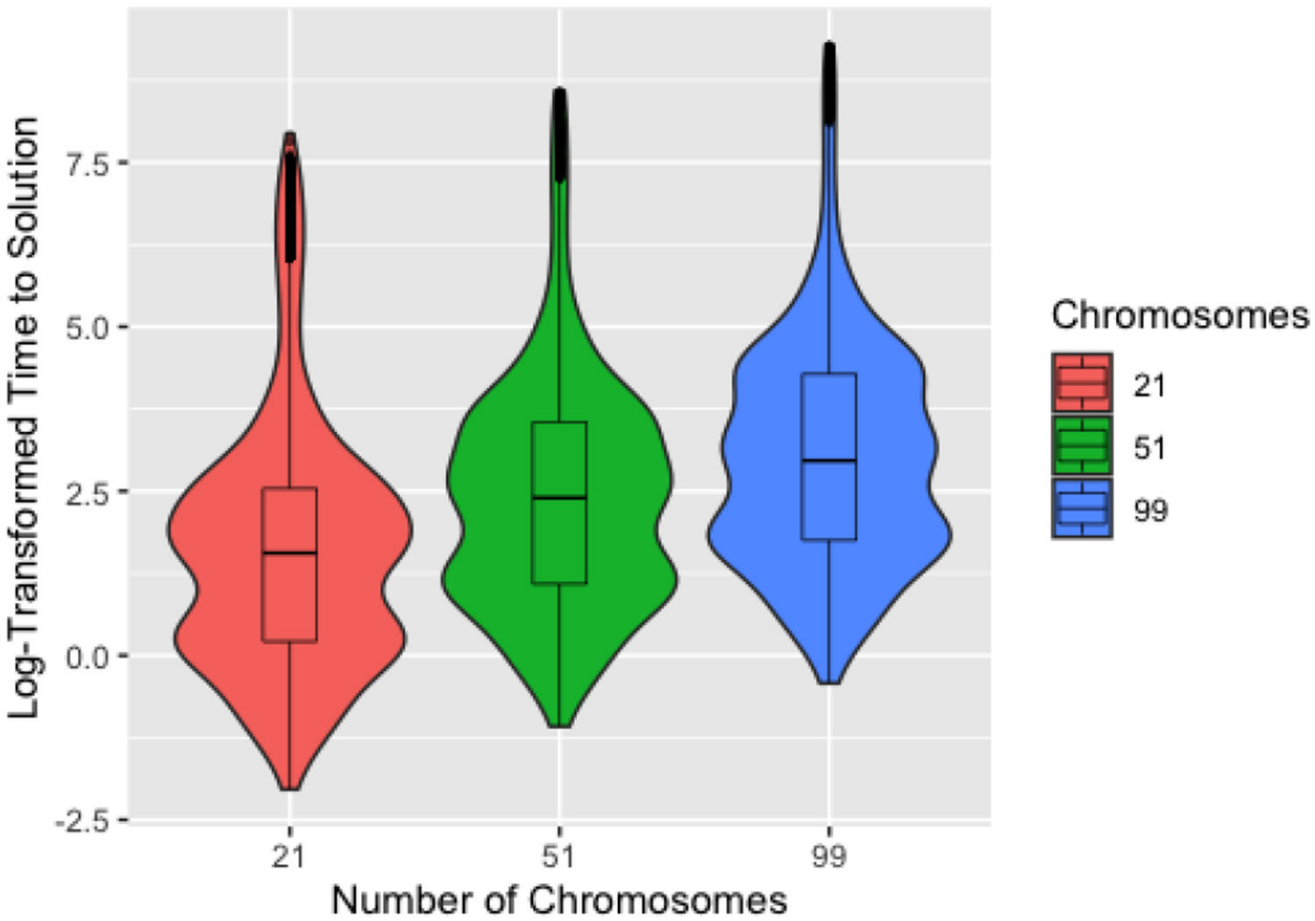
Boxplots and violin plots for log time to identify a solution (in seconds) by number of chromosomes for 200 generations without improvement.

Figure 7 shows the time to identify a solution on the log scale by number of chromosomes, with median times to identify a solution of 4.7 s, 11.0 s, and 19.3 s for 21, 51, and 99 chromosomes, respectively. This demonstrates that, for the computer used to conduct the simulation study, the efficiency of using fewer chromosomes outweighs any potential reduction in number of generations from using more chromosomes. Since the number of chromosomes used—after excluding 5—had little effect on the number of generations required to identify a solution, we expect this to be true for most users. It should also be noted that, while the magnitude of the differences in time are substantial on the scale used, these differences are fairly negligible in practice. The exception to this is for conditions that are known to increase computation time, such as large and high-degree networks. Since computational time scales roughly linearly with the number of chromosomes (*O*(*n*) complexity), using a high number of chromosomes can substantially increase computation time under these conditions.

## 4. Discussion

While we discuss the following specifically in the context of the opinion diffusion application, the hyperparameters of concern are the result of a parameter space with many perfect solutions other than the parameters used to generate the data. The behavior and suggestions for mitigation, along with the associated operator modifications, are relevant to other applications of genetic algorithms under similar conditions. Overall, the algorithm is fairly robust to the hyperparameter values selected, with number of generations without improvement (iterb, iterc, iterm, iters, and iterr) and number of chromosomes (chromosomes) being notable exceptions. We recommend using at least 21 chromosomes, though using more should have minimal practical impact on computation time, except in cases where the networks are large—increasing the size of the chromosomes—or more dense—making the chromosomes less sparse. For the hyperparameters in the ProbSigma, MinMax, and MultFactor groupings, we suggest values close to those in the *medium* and *moderate* levels simply because they fall roughly in the center of ranges of values demonstrated to perform well. The exception to this suggestion is when users may seek to use these hyperparameters to mitigate undesirable effects from the number of generations without improvement.

The results suggest using 200 generations without improvement is a good starting point for all relevant hyperparameters because of both the performance in recovering parameters and the low number of generations typically needed to find a solution. While the number of generations to identify a solution may increase in practical applications—without a guaranteed solution and with agents missing from the network—the user will receive this feedback and can adjust accordingly. We identified the bias against edge cases inherent in our weight matrix generation process as an explanation for the poor parameter recovery for runs using either 1000 or 5000 generations without improvement, pointing to iters—which triggers the change to the control parameter *σ* within the mutation operator—as the hyperparameter of concern. The choice of itetrs is the one decision where we encourage caution and careful consideration, particularly because the consequences are not just poor efficiency but also poor recovery.

While the bias against edge cases is clear in the networks used in this simulation study, the extent to which this is a concern for real-world opinion diffusion processes is unknown. Networks of stubborn individuals would be biased toward the boundary, while networks of highly receptive individuals could be biased either toward or away from the boundary, depending on whether they have preexisting opinions on the topic. Unfortunately, it is not possible to distinguish between these cases using the opinion data since consistent opinions across time could indicate either stubborn individuals or receptive individuals only connected to those with similar opinions. As such, it would be irresponsible to intentionally direct the algorithm toward or away from the boundaries using the hyperparameter. Instead, the user must find a balance between forcing the algorithm to search only the boundaries or beginning the exploitation phase without first exploring the boundaries. Recall that, since the method for generating the initial chromosomes is also biased against edge cases, setting the initial probability of mutation (probm) to zero or making the initial value of the control parameter *σ* (sigma) very small is not a viable solution, avoiding concerns about becoming stuck at the boundary by preventing the algorithm from exploring them at all.

As with the other hyperparameters controlling the number of generations without improvement before the control parameters are modified and the elite chromosome reintroduced, our recommendation for finding this balance for iters is to test different values and make modifications based on the feedback. Users can decrease the value of iters if the algorithm consistently identifies solutions at the boundary or increase iters to ensure they are being searched. A value closer to one for factors can also be used to control how quickly the algorithm moves away from the boundaries, mitigating the choice of an inappropriately low value of iters. Since the number of generations without improvement must be reached for factors to be relevant, this is not an option for correcting inappropriately high values of iters. Though not directly tied to the hyperparameters, using more time steps or a more precise scale can minimize the effect of iters by decreasing the prevalence of perfect solutions with poor recovery, which we already suggest as they improve overall performance of the method.

In summary, we suggest at least 21 chromosomes, values close to the *medium* and *moderate* levels for the ProbSigma, MinMax, and MultFactor groupings, and setting iterb, iterc, iterm, iters, and iterr to 200 as initial values. Users should assess performance with these values and make modifications as necessary. Since inappropriate values of iters inhibit a proper search of the parameter space, especially when used with a high value of sigma, we strongly recommend paying close attention to this hyperparameter. In cases where forcing a search of only the boundaries is of particular concern, such as datasets with limited time steps and imprecise ordinal scales, users can use a conservative (low) value of iters, mitigating concerns about failing to explore the edge cases by using values of factors closer to one. While all the discussion surrounding iters may seem intimidating, we want to highlight that the algorithm is robust to the choices of all but a few hyperparameter values, all of which are discussed here and for which initial, if not default, values are suggested.

## Figures and Tables

**Table 1.** Name and description of all hyperparameters used in the algorithm.

Hyperparameter	Description
chromosomes	Number of chromosomes
probb	Initial probability of blending (*p_b_*)
factorb	Multiplicative factor for modifying *p_b_*
maxb	Maximum value of *p_b_*
iterb	Number of iterations with no improvement before modifying *p_b_*
probc	Initial probability of crossover (*p_c_*)
factorc	Multiplicative factor for modifying *p_c_*
minc	Minimum value of *p_c_*
iterc	Number of iterations with no improvement before modifying *p_c_*
probm	Initial probability of blending (*p_m_*)
factorm	Multiplicative factor for modifying *p_m_*
minm	Minimum value of *p_m_*
iterm	Number of iterations with no improvement before modifying *p_m_*
sigma	Initial value of standard deviation *σ* of *ε* for mutation operator
factors	Multiplicative factor for modifying *σ*
mins	Minimum value of *σ*
iters	Number of iterations with no improvement before modifying *σ*
max_iter	Maximum number of iterations to run algorithm
min_improve	Minimum decrease in value of objective function considered an improvement
min_dev	Acceptable value of objective function for stopping algorithm
reintroduce	Type of chromosome to be reintroduced
iterr	Number of iterations with no improvement before reintroducing chromosome

**Table 2.** Grouping levels and hyperparameter values for ProbSigma.

Level	probb	probc	probm	sigma
Low	0.01	0.05	0.05	0.2
Medium	0.1	0.1	0.1	0.5
High	0.2	0.2	0.2	1

**Table 3.** Grouping levels and hyperparameter values for MinMax.

Level	maxb	minc	minm	mins
Minimal	1	0	0	0
Moderate	0.5	0.01	0.01	0.01
Extreme	0.2	0.05	0.05	0.05

**Table 4.** Grouping levels and hyperparameter values for MultFactor.

Level	factorb	factorc	factorm	factors
Slow	2	0.5	0.5	0.5
Moderate	5	0.2	0.2	0.2
Rapid	10	0.1	0.1	0.1

**Table 5.** Inputs used in the hyperparameters simulation study.

Input	Values	Notes
Network Size	*N* = 4, 20, 50	reachability enforced
Mean Degree	*d* = 2, 5, 9	minimum degree *d* = 1 for all nodes
Self-weight	*w_ii_* = 0.5	beta distribution with *κ* = *α* + *β* = 4
Time Steps	*T* = 2, 3, 6	
Scale Bins	*n* = 5, 7, 10, 20, 30	
Chromosomes	5, 21, 51, 99	chromosomes hyperaprameter
ProbSigma	low, medium, high	(see Table 2)
MinMax	minimal, moderate, extreme	(see Table 3)
MultFactor	slow, moderate, rapid	(see Table 4)

## Data Availability

The data generated and analysed during the simulation study are available in the file “hyper.csv.zip” in the corresponding author’s GitHub repository: https://github.com/karajohnson4/DeGrootGeneticAlgorithm. The genetic algorithm code is also available in the corresponding author’s GitHub repository under the name Algorithm-Code. The Algorithms-archive branch will serve as an archived version. The code is written in Julia, is platform independent, requires Julia 1.5 or higher, and uses the GNU GENERAL PUBLIC LICENSE [25].

## Abbreviations

The following abbreviations are used in this manuscript:

SODM: stochastic opinion dynamics model
BFO: bacterial foraging optimization
PSO: particle swarm optimization
SCBFO: self-adaptive chemotaxis strategy bacterial foraging optimization
CRS: Chess Rating System
REVAC: Relevance Estimation and Value Calibration
RMSE: root-mean-square error
